# Quantifying the Importance of Abiotic and Biotic Factors Governing the Succession of Gut Microbiota Over Shrimp Ontogeny

**DOI:** 10.3389/fmicb.2021.752750

**Published:** 2021-10-08

**Authors:** Wenqian Zhang, Zidong Zhu, Jiong Chen, Qiongfen Qiu, Jinbo Xiong

**Affiliations:** ^1^State Key Laboratory for Managing Biotic and Chemical Threats to the Quality and Safety of Agro-Products, Ningbo University, Ningbo, China; ^2^School of Marine Sciences, Ningbo University, Ningbo, China; ^3^School of Biochemical Engineering, Jingzhou Institute of Technology, Jingzhou, China

**Keywords:** shrimp gut microbiota, bacterioplankton community, temporal succession, SourceTracker, ecological processes, rare and abundant sub-communities

## Abstract

Intensive studies have evaluated abiotic factors in shaping host gut microbiota. In contrast, little is known on how and to what extent abiotic (geochemical variables) and biotic (i.e., surrounding microbes, younger shrimp, and age) factors assemble the gut microbiota over shrimp ontogeny. Considering the functional importance of gut microbiota in improving host fitness, this knowledge is fundamental to sustain a desirable gut microbiota for a healthy aquaculture. Here, we characterized the successional rules of both the shrimp gut and rearing water bacterial communities over the entire shrimp farming. Both the gut and rearing water bacterial communities exhibited the time decay of similarity relationship, with significantly lower temporal turnover rate for the gut microbiota, which were primarily governed by shrimp age (days postlarval inoculation) and water pH. Gut commensals were primary sourced (averaged 60.3%) from their younger host, rather than surrounding bacterioplankton (19.1%). A structural equation model revealed that water salinity, pH, total phosphorus, and dissolve oxygen directly governed bacterioplankton communities but not for the gut microbiota. In addition, shrimp gut microbiota did not simply mirror the rearing bacterioplankton communities. The gut microbiota tended to be governed by variable selection over shrimp ontogeny, while the rearing bacterioplankton community was shaped by homogeneous selection. However, the determinism of rare and stochasticity of abundant subcommunities were consistent between shrimp gut and rearing water. These findings highlight the importance of independently interpreting host-associated and free-living communities, as well as their rare and abundant subcommunities for a comprehensive understanding of the ecological processes that govern microbial successions.

## Introduction

*Litopenaeus vannamei* is one of the most valuable shrimp species in aquaculture globally, while its production is being threatened by diverse diseases. It is now widely recognized that the gut microbiota contributes indispensable roles in sustaining host health (Xiong, 2018). For this reason, intensive studies have focused on factors shaping the gut microbiota, including life stage (i.e., larva, juvenile, or adult), disease (Lu et al., 2022), and surrounding environmental factors (Xiong et al., 2017; Hou et al., 2018). In contrast, we know little about how the biotic sources, e.g., younger host, affect the shrimp gut microbiota.

It is supposed that aquatic animals have a close association with their surrounding water microbiomes (De Schryver et al., 2014). However, survey studies show that the gut microbiota of shrimp is distinct from that in rearing water and/or sediment (Huang et al., 2018; Song et al., 2020). Our recent work evaluates to what extent rearing water and sediment bacterial communities affect the gut microbiota of shrimp, illustrating that shrimp acquire little of their gut commensals from rearing water (Xiong et al., 2019b). Instead, 66.7% gut commensals of the adult shrimp are derived from their juveniles (Zhang et al., 2021). In accordance, ample evidence has shown that the structures of gut microbiota are differed significantly along shrimp life stages, which are distinct from those in the rearing water (Burns et al., 2016; Yan et al., 2016; Zhang et al., 2018). In this context, temporal changes in the shrimp gut microbiota are not parallel with those in rearing bacterioplankton. However, it seems that the gut microbiota of larval shrimp is more similar with the rearing bacterioplankton community, compared with the adults (Burns et al., 2016; Xiong et al., 2018). A possible explanation is that the selection on external taxa is increased as host matured (Xiong et al., 2019a; Xiao et al., 2021). However, the deteriorated water quality imposes stress on shrimp, which in turn depresses their capability of filtering on external taxa (Xiong et al., 2017). For example, nutrient accumulation in rearing water significantly alters shrimp gut microbiota at later farming stage (Lucas et al., 2010; Zhang et al., 2014; Xiong et al., 2016). Accordingly, there is non-linear trend in the relative importance of deterministicity in governing the gut microbiota over shrimp development (Xiong et al., 2019b). It is now recognized that the gut microbiota is conjointly affected by rearing geochemical variables, bacterioplankton, and host development (Xiong et al., 2019b; Xiao et al., 2021), while little is known on the interplay among these variables. Theoretical evidence has proposed that the successional pattern of host-associated (e.g., gut microbiota) communities is distinct from that of free-living bacteria (e.g., bacterioplankton) (Baselga, 2010; Xiong et al., 2019b), whereas experimental evidence is lacking. For these reasons, it remains unclear how and to what extent the gut microbiota is affected by rearing bacterioplankton community as shrimp aged, whereas this knowledge is fundamental for sustaining a health aquaculture.

A microbial community is comprised by a large number of rare species and a few highly abundant taxa (Brown et al., 2014). It is becoming clear that rare biosphere is functionally and ecologically important in a given community (Lynch and Neufeld, 2015). For example, rare taxa serve a reservoir that can quickly respond to environmental changes, thereby promoting community stability in a wide variety of ecosystems (Shade et al., 2014). Additionally, rare subcommunity also contributes dispensable roles in nutrient cycling (Pester et al., 2010). Available studies have depicted that the freeing-living rare and abundant communities exhibit contrasting assembly processes (Mo et al., 2018). However, it remained uncertain whether the host-associated counterparts are ruled by the same ecological processes, as what has been observed for freeing-living community.

An ultimate goal of microbial ecology is to predict the responses of microbial communities to changing environments, yet this goal is difficult to achieve. One reason for this challenge is that there are two types of ecological processes, deterministicity and stochasticity, governing the microbial assembly (Van Der Gast et al., 2008). Deterministic processes include abiotic/biotic selection and biological interaction, while stochastic processes (also known as neutral processes) include dispersal-related processes and ecological drift (Venkataraman et al., 2015; Zhou and Ning, 2017). It has been perceived by ecologists that both deterministic and stochastic processes occur simultaneously in assembling local communities (Chase, 2010; Zhou et al., 2014), whereas no consensus has emerged regarding their relative importances. For a given community, if it is tailored by the dominance of deterministic processes, the temporally successional trend is predictable (Vanwonterghem et al., 2014). Intriguingly, it has been shown that the degrees of deviation in the gut microbiota from the successional trajectory as host aged are positively associated with the severity of the shrimp disease (Xiong et al., 2015). In this regard, it is essential to explore the underlying ecological processes governing the succession of gut microbiota over shrimp ontogeny.

Herein, we explored the successional rules of both the gut and the rearing water bacterial communities over the entire shrimp farming. The main purposes were (1) to evaluate the interplay among biotic (shrimp age), abiotic (water geochemical variables) factors, bacterioplankton community, and the shrimp gut microbiota; (2) to quantify the relative importances of external and internal sources to the gut microbiota over shrimp ontogeny; (3) to compare the underlying ecological processes governing the shrimp gut and rearing water bacterial communities, including total, abundant, and rare communities, by integrating multiple ecological approaches.

## Materials and Methods

### Experimental Design and Sample Collection

Larval shrimp (*L. vannamei*) were introduced into 60 identical greenhouse ponds (concrete and rectangular, 30 m × 60 m, with a depth of 1.2 m) on April 8, at Zhanqi, Ningbo, eastern China (29°32′N, 121°31′E). One week later, both shrimp and rearing water samples were collected with an interval of 6–10 days from six selected ponds over the entire shrimp farming (from 15 April to 10 July). In order to remove microorganisms and suspended particles, rearing seawater was disinfected with sodium hypochlorite and alum and then aerated in open reservoirs for 3 weeks before usage. To reduce the spatial variability, water samples were collected from four representative points (similar locations in all ponds) and then pooled to compose one biological sample for a given pond. Water samples were stored in an icebox and were transported to laboratory for further processing. In total, we collected 144 samples (6 replicates × 12 samplings × 2 habitats) for microbial community analysis.

Water temperature (WT), pH, salinity (SAL), and dissolved oxygen (DO) were recorded *in situ* using corresponding probes (Oxi 340i; WTW, Weilheim, Germany) at a depth of 50 cm (below water surface). The concentrations of water total phosphorus (TP) and total nitrogen (TN) were analyzed following seawater analysis standard of China (AQSIQ, 2007).

### DNA Extraction, Amplification, and Sequencing

To collect microbial cells, 0.5 L of water sample was prefiltered through nylon mesh (100 μm pore size) and subsequently filtered onto a 0.22-μm membrane (Millipore, Boston, MA, United States) on the sampling day. To obtain high efficiency of DNA extracts, the pooled number of shrimp individuals was decided on the basis of their intestine size. Specifically, every three, two, or one intestine from larval, juvenile, or adult shrimp was pooled to compose one biological sample for each pond, respectively. The filters and shrimp intestines were placed into sterile tubes and were stored at –80°C.

Genomic DNA (gDNA) was extracted using a FastDNA Spin kit (MP Biomedicals, Carlsbad, CA, United States) following the manufacturer’s protocols. The V3–V4 regions of bacterial 16S rRNA gene were amplified by primers: 341F (5′−CCTAYGGGRBGCA-SCAG−3′) and 806R (5′−GGACTACNNGGGTATCTAA-3′). For each sample, triplicate 50 μl PCRs were performed which contained 25 ng DNA extracts as template with the following conditions: 25 cycles of denaturation at 95°C for 30 s, annealing at 55°C for 30 s, and extension at 72°C for 45 s, with a condition of 72°C for 10 min for the final elongation step. The triplicate amplicons for each sample were pooled and purified using a PCR fragment purification kit. Equimolar amounts of amplicons from each sample were pooled and then were sequenced in a single run using the Illumina MiSeq platform (Illumina, San Diego, CA, United States), resulting in 2 × 300 bp paired-end reads.

### Processing of Illumina Sequencing Data

The paired-end reads were joined and assigned to samples based on barcode. The merged sequences were analyzed using the QIIME2 pipeline (Caporaso et al., 2010). Specifically, sequences at < 200 bp in length, showed ambiguous bases, or had a mean quality score < 20 were filtered. Then, sequences were binned into operational taxonomic unit (OTU) with 97% cutoff using UCLUST (Edgar, 2010). The most abundant sequence from each OTU was selected as representative and then was taxonomically assigned a closed reference (Greengenes Database, release 13.8) (DeSantis et al., 2006), which enables each identified OTU to have a close relative. After the taxonomy had been assigned, Archaea, Chloroplast, unclassified Bacteria, as well as singletons, were excluded from subsequent analysis.

### Statistical Analysis

We defined OTUs with a mean relative abundance of ≥ 0.01% across the samples as “abundant” OTUs, whereas OTUs with a mean relative abundance of < 0.001% as “rare” OTUs follow the criterion as described elsewhere (Logares et al., 2014; Liu et al., 2015).

All statistical analyses were performed in the R-environment^1^ unless otherwise indicated. To improve normality and homoscedasticity, bacterial communities were Hellinger transformed, while environmental variables were normalized by using function decostand in package vegan. Heatmap was used to depict the abundance of the top 20 dominant bacterial genera in the shrimp gut microbiota and those in the bacterioplankton communities. Paired *t*-test (pond served a conditional factor) was used to evaluate the significance (*p* < 0.05 level) of diversity between gut and corresponding water bacterial communities at each sampling. A non-metric multidimensional scaling (NMDS) analysis was used to compare the differences in the structures of rearing water and shrimp gut bacterial communities based on Bray-Curtis distance. The significance between groups was tested using an analysis of similarity (ANOSIM) (Anderson, 2010). Permutational multivariate analysis of variance (perMANOVA) was conducted to quantify the relative contributions of habitat (gut or water), shrimp age (days postinoculation), and their interaction to the variations in bacterial community using the “adonis” function (Anderson, 2010).

The time decay of similarity relationship was used to compare the temporal turnover rate (the slope of the regression) between the gut and water bacterial communities over shrimp farming (Xiong et al., 2014). To account for zero similarity values, bacterial community similarity and lag of shrimp age were ln transformed (Talbot et al., 2014). Here, we treated pond as a conditional factor, thereby enabling us to compare the significance (paired *t*-test) in turnover rate between gut and rearing water communities. The multiple regression on distance matrices (MRM) was further used to determine variables that triggered the temporal turnover of bacterial communities. This approach offers advantages over the traditional partial Mantel test to investigate linear, non-linear or non-parametric relationships between a multivariate response distance matrix and any number of explanatory distance matrices (Legendre et al., 1994; Lichstein, 2007). To minimize the collinearity between environmental factors, we used variable clustering to assess the redundancy of variables by the “VARCLUS” procedure in package Hmisc before applying MRM. Then, a matrix randomization procedure with standardized predictor variables was implemented using package ecodist (Goslee and Urban, 2007). To reduce the effect of spurious relationships between variables, we ran the MRM test twice, after removal of insignificant variables by the first run. The results were reported from the second run. A structural equation model (SEM) was used to uncover the interplay among rearing water geochemical variables, bacterioplankton and gut bacterial communities, and shrimp age in AMOS 23.0 (IBM, Chicago, IL, United States) (Byrne, 2001).

SourceTracker analysis was employed to quantify the relative contributions of both external (rearing water bacterioplankton community) and internal (the gut microbiota of adjacent younger shrimp) resources to the shrimp gut microbiota (Knights et al., 2011). This approach analyzed the relative abundance of each OTU share in water or younger shrimp gut with older ones, to calculate the probability that each OTU detected in the shrimp gut was sourced from the rearing water or adjacent younger shrimp.

To evaluate the phylogenetic community assembly, the “standardized effect size” of phylogenetic community structure (ses.MNTD) was calculated for non-random phylogenetic relatedness (MNTD) by the difference between phylogenetic distances in the observed communities vs. null communities generated with 999 randomizations, divided by the standard deviation of phylogenetic distances in the distribution using the Picante package (Kembel et al., 2010; R Core Team, 2013). For a given community, ses.MNTD value less than –2 indicates that the community is more phylogenetically related than expected by chance (determinism), whereas ses.MNTD value greater than +2 indicates that a community is less closely related than expected by chance (stochasticity) (Webb et al., 2002; Stegen et al., 2012). Pairwise phylogenetic turnover between communities was calculated as the MNTD metric (βMNTD) using the “comdistnt” function (abundance.weighted = TRUE) in package picante (Kembel et al., 2010). The community assembly processes were further evaluated by βNTI using the “ses.mntd” function (Kembel et al., 2010) and a null modeling approach (Stegen et al., 2012), respectively. βNTI (the difference between the calculated βMNTD and the null-model estimation) values were quantified by either accounting for βNTI is the number of standard deviations that the observed βMNTD is from the mean of the null distribution. A value of βNTI of > 2 or < –2 indicates greater than or less than the expected phylogenetic turnover, respectively (Stegen et al., 2012).

## Results

### Temporal Successions of Shrimp Gut and Rearing Water Bacterial Communities

**FIGURE 1:**
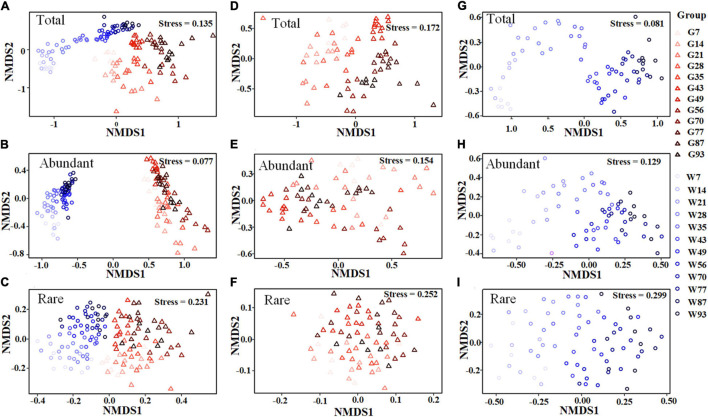
Non-metric multidimensional scaling (NMDS) ordinations showing the structures of total, abundant and rare communities. NMDS ordination of the total, abundant, and rare bacterial communities in both the shrimp gut and rearing water **(A–C)**, shrimp gut **(D–F)**, rearing water **(G–I)** based on Bray-Curtis similarity, respectively. Samples were coded and colored by habitat and shrimp life stage, of which G and W indicate shrimp gut and water, respectively.

**FIGURE 2:**
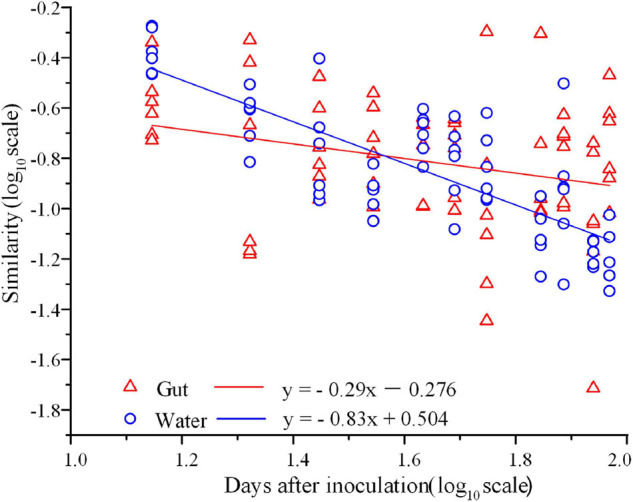
Time-decay relationship for shrimp gut microbiota and bacterioplankton communities. The *x*-axis is log in days postlarval shrimp inoculation, and *y*-axis is log (similarity) calculated using the Bray-Curtis distance (*R* = 0.407, *p* = 0.001).

In total, 3,842,244 high-quality sequences, with 17,385–36,579 sequences per sample (mean ± standard deviation, 26,868 ± 4,620) were collected across the enrolled 143 samples. After rarefaction, we obtained 32,811 OTUs in the analysis. Diversity of the gut microbiota was temporally stable over shrimp ontogeny, whereas bacterioplankton diversity linearly increased over the same timeframe, as supported by both Shannon and phylogenetic diversity indices (Supplementary Figure 1). The NMDS biplot depicted that the gut microbiota were distinct from bacterioplankton communities (Figure 1). There were sequential successions of both the gut microbiota and bacterioplankton communities during shrimp farming, as evidenced by increased distances along NMDS axis 1 (Figure 1). These differences were confirmed by the ANOSIM, illustrating that shrimp gut bacterial communities were significantly distinct between every paired age (Supplementary Table 1). In contrast, there were no significant differences between some adjacent pairs of bacterioplankton communities, e.g., W28 vs. W21, W56 vs. W49, W77 vs. W70, and W93 vs. W87 (Supplementary Table 2). Furthermore, perMANOVA revealed that both habitat (shrimp gut or rearing water) and shrimp age significantly (*p* < 0.001 in both cases) contributed to the variations in bacterial community. Shrimp age exerted consistently higher importances than habitat in governing the total, abundant, and rare bacterial communities (Table 1). Both the gut microbiotas and bacterioplankton communities exhibited significant time decay of similarity relationship. The temporal turnover rate of bacterial community in shrimp gut (–0.290 ± 0.127) was significantly (paired *t*-test, *p* = 0.001) lower than that in rearing water (–0.827 ± 0.083) (Figure 2). MRM revealed that the temporal succession was primarily governed by shrimp age, water temperature, and pH (Table 2). Notably, each of the three variables exerted higher contributions in governing the bacterioplankton communities compared with the gut microbiota (Table 2), suggesting that bacterioplankton communities were more strongly affected by environmental factors. The MRM model explained 66% (*p* < 0.001) variation in bacterioplankton community, while only 21% (*p* < 0.001) in the gut microbiota. Additionally, shrimp age contributed larger partial regression coefficients in shaping abundant subcommunities than corresponding rare counterparts in both the gut and rearing water.

**TABLE 1:** Quantitative effects of sampling time and habitats on variation in community composition using non-parametric permutational multivariate analysis of variance (perMANOVA) with adonis function.

	**Age**		**Habitats**		**Age:habitats**	
	***R* ^2^**	***P***	***R* ^2^**	***p***	***R* ^2^**	***p***
Community structure						
Total	0.306	<0.001	0.150	<0.001	0.431	<0.001
Rare	0.116	<0.001	0.022	<0.001	0.138	<0.001
Abundant	0.315	<0.001	0.167	<0.001	0.455	<0.001

*The R^2^ values represent the proportion of the community variation constrained by each variable or their interaction.*

**TABLE 2:** Results of the multiple regression on distance matrices (MRM) for shrimp gut, bacterioplankton, and their abundant and rare communities.

**Habitat**	**Gut**						**Rearing water**					
	**Total**		**Rare**		**Abundant**		**Total**		**Rare**		**Abundant**	
	***R*^2^ = 0.21**	***p* < 0.001**	***R*^2^ = 0.03**	***p* < 0.001**	***R*^2^ = 0.15**	***p* < 0.001**	***R*^2^ = 0.66**	***p* < 0.001**	***R*^2^ = 0.27**	***p* < 0.001**	***R*^2^ = 0.65**	***p* < 0.001**
Age	0.181	<0.001	0.007	0.001	0.118	<0.001	0.421	<0.001	0.229	<0.001	0.541	<0.001
WT	0.093	0.01	ND	ND	0.089	<0.001	0.301	<0.001	ND	ND	0.299	<0.001
pH	0.029	0.04	0.009	0.002	0.024	0.008	0.364	<0.001	ND	ND	0.358	<0.001
SAL	NS	NS	ND	ND	ND	ND	0.514	<0.001	0.198	<0.001	0.5	<0.001
BOD	NS	NS	ND	ND	ND	ND	0.216	0.002	ND	ND	0.216	<0.001
DO	ND	ND	ND	ND	ND	ND	0.258	<0.001	0.13	<0.001	0.256	<0.001
TP	ND	ND	0.014	<0.001	ND	ND	ND	ND	0.076	<0.001	ND	ND
TN	ND	ND	0.001	NS	ND	ND	0.254	<0.001	ND	ND	ND	ND

*ND, not determined (removed by the VARCLUS results); NS, not significant.*

### Factors Governing the Temporal Successions of Bacterial Community

**FIGURE 3:**
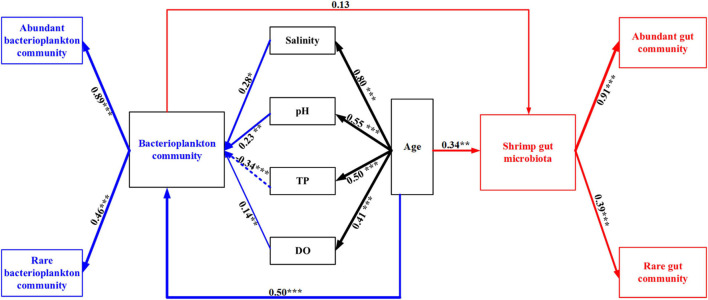
Structural equation modeling (SEM) shows the effect of environmental factors and bacterioplankton community on shrimp gut community. The numbers on arrows indicate standardized path coefficients. Arrow widths show the strength of the causal relationship. Arrows in blue and red indicate the effects on the bacterioplankton community and the shrimp gut microbiota, respectively. Solid and dashed lines indicate positive and negative correlations, respectively. *p < 0.05, **p < 0.01, ***p < 0.001.

A forward selection procedure identified four water variables (TP, DO, pH, salinity) and shrimp age that significantly contributed to the variations in bacterial community (*p* < 0.01) (Supplementary Figure 2 and Supplementary Table 3). The four water variables were significantly associated with shrimp age, which were attributed to the temporal dynamics of water variables during shrimp farming. Bacterioplankton community was positively correlated with salinity (λ = 0.28, *p* = 0.007), pH (λ = 0.23, *p* = 0.002), and DO (λ = 0.14, *p* = 0.048), and was negatively affected by TP (λ = –0.34, *p* < 0.001) (Figure 3 and Supplementary Table 3). Shrimp age was significantly associated with bacterioplankton community (0.50). The gut microbiota was affected by the combination of direct (0.34) and weak indirect (0.09) effects of shrimp age (Figure 3 and Supplementary Table 3). Notably, bacterioplankton community contributed a weak and insignificant direct effect on the assembly of shrimp gut microbiota. As expected, abundant and rare subcommunities exhibited significant and positive contributions to corresponding total bacterial communities, with much higher contributions of the abundant subcommunities (Figure 3 and Supplementary Table 3).

### Sources of Shrimp Gut Commensals Over Shrimp Ontogeny

**FIGURE 4:**
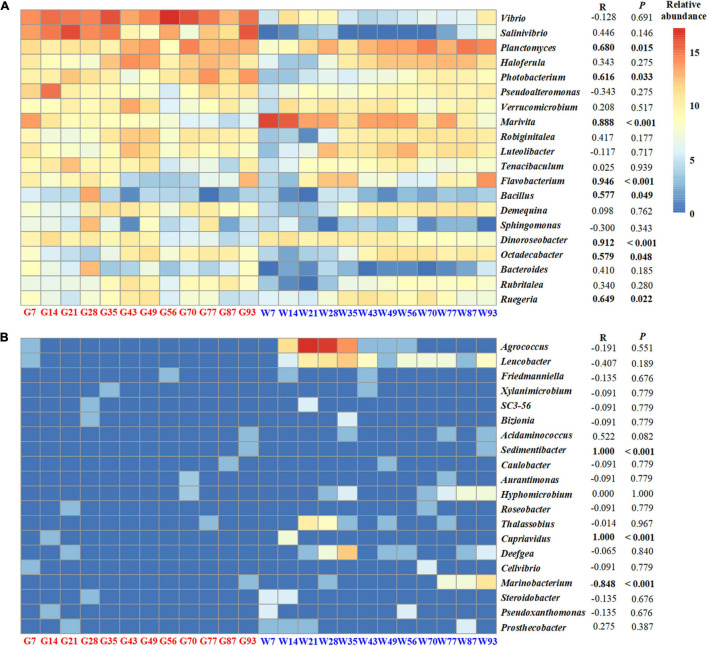
The relative abundances of the top abundant 20 bacterial genera **(A)** and the most rare 20 bacterial genera **(B)** in the gut and those in bacterioplankton communities. Pearson’s correlations and the significances of the genera between shrimp gut and rearing water are shown on the right.

The relative proportion of shared OTUs between shrimp gut and rearing water was negligible, ranged from 0.26 to 2.11% (Supplementary Figure 3). Thus, the rearing water bacterioplankton community contributed minor role in affecting the shrimp gut microbiota. To test whether gut microbiota parallelly changed with rearing water bacterial communities along shrimp farming, temporal dynamics of the top 20 dominant bacterial genera in the shrimp gut were compared with those in the rearing water (Figure 4). The relative abundances of *Vibrio*, *Salinivibrio*, and *Haloferula* genera were abundant in shrimp gut but were rare in rearing water. Only six dominant bacterial genera in shrimp gut, such as *Ruegeria*, *Marivita*, and *Flavobacterium*, were positively correlated with these in bacterioplankton communities, but not for the other 12 genera, including *Vibrio* and *Pseudoalteromonas* (Figure 4A). A similar pattern was observed for the most rare 20 bacterial genera in the shrimp gut, in which only genera of *Sedimentibacter*, *Cupriavidus*, and *Marinobacterium* were significantly associated with these in rearing water (Figure 4B). Overall, the compositions and abundances of the bacteria in gut were insignificantly affected by the rearing bacterioplankton community over shrimp ontogeny.

**FIGURE 5:**
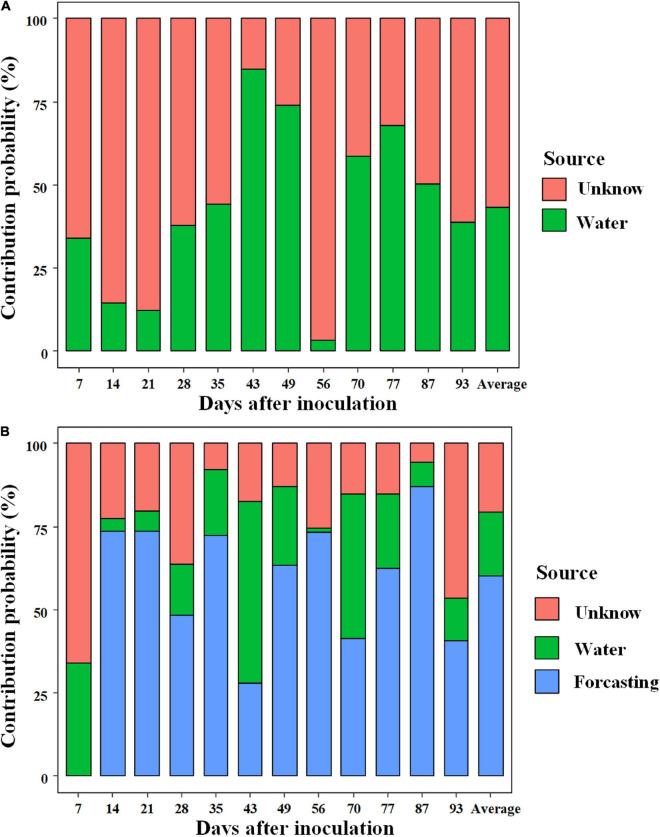
SourceTracker analyzes the relative contribution of external (rearing water) **(A)** and internal (gut commensals of the adjacent younger shrimp) sources **(B)** to the shrimp gut commensals.

SourceTracker analysis was used to quantify the relative contribution of external sources (rearing water) on the shrimp gut microbiota at each sampling (Figure 5A). In general, larval shrimp (breeding days less than 35) sourced little commensals from rearing water compared with juveniles and adults, with the exception on day 56. Bacterioplankton community contributed 43.3% (averaged contribution) of the species to shrimp gut microbiota, whereas most of the source was unknown (Figure 5A). When integrating the adjacent younger shrimp as an internal source for the gut microbiota in the model, the relative contribution of rearing water to gut microbiota sharply decreased to 19.1% (averaged proportion, ranged from 1.22 to 54.7%). Instead, gut commensals were primarily derived from the adjacent younger shrimp, with an averaged contribution of 60.3%. Accordingly, the proportion of unknown source of gut microbiota sharply decreased to 20.5% (Figure 5B). Taken together, the majority of gut commensals sourced little species from surrounding species pool, which were temporally sustained over shrimp ontogeny.

### Ecological Processes Govern the Successions of Bacterial Community

**FIGURE 6:**
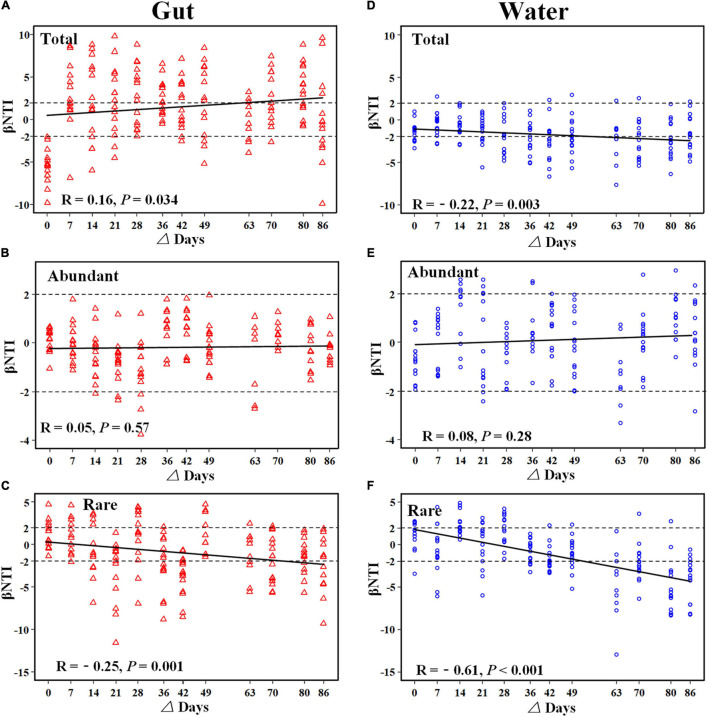
Relationship between βNTI values of bacterial community over shrimp ontogeny. The horizontal dashed lines indicate the βNTI values of –2 and +2. An individual community below or above the two dashed lines indicates that determinism dominantly governs the community assembly, while between the two dashed lines indicates that stochasticity is dominant. The relationship between βNTI and differences in days gut microbiota **(A–C)** and bacterioplankton community **(D–F)** was fitted using linear regression.

The ses.MNTD values of gut and rearing water bacterial communities were significantly lower than zero, suggesting that the two communities tended to be phylogenetically clustering (Supplementary Figure 4). Additionally, most of the βNTI values of gut microbiotas and bacterioplankton communities were less than –2, indicating that the dominant role of deterministic processes assembled the gut microbiota and bacterioplankton community (Supplementary Figure 5). To evaluate the trends of ecological processes over shrimp ontogeny, βNTI values were regressed against the lag of shrimp age. The temporal trends of βNTI were different between shrimp gut and rearing water bacterial communities (Figure 6). Specifically, there was a significant and positive correlation (*R* = 0.16, *p* = 0.034) between βNTI values of the total gut microbiota as shrimp aged (Figure 6A), whereas those of total bacterioplankton community exhibited the opposing trend (*R* = –0.22, *p* = 0.003) (Figure 6B). There were no significant correlations between βNTI values of the abundant subcommunities in gut or rearing water during shrimp farming. In addition, most of the βNTI values of abundant subcommunity were between –2 and 2, indicating the dominant role of stochastic processes in assembling abundant subcommunity (Figures 6C,D). In contrast, the βNTI values of rare subcommunities in both the gut (*R* = –0.25, *p* = 0.001) and rearing water (*R* = –0.61, *p* < 0.001) significantly decreased over the same timeframe, which tended to be less than –2 (Figures 6E,F).

## Discussion

Despite recent progress, little is known about the underlying ecological processes governing the successional patterns of host-associated microbes, especially their abundant and rare counterparts. To address this pressing knowledge gap, we explored how and to what extent abiotic (water geochemical variables) and biotic (i.e., younger shrimp, host age, and rearing bacterioplankton community) factors affected the gut microbiota over shrimp ontogeny. In addition, we quantified the relative contributions of external (rearing water) and internal (adjacent younger shrimp gut microbiota) sources on the shrimp gut commensals. These findings yield novel insights into the assembly of gut microbiota over shrimp ontogeny from an ecological perspective. It is worthy to note that we used OTU clustering methods instead of the more recently developed amplicon sequence variants (ASVs). However, it has been shown that all α and β diversity metrics are highly positively correlated (*r* > 0.90) between samples analyzed with either ESVs or traditional OTUs. ESV or OTU methods often reveal similar ecological results, with indistinguishable statistical inferences (Glassman and Martiny, 2018). Similarly, a recent study depicts that OTUs and ASVs produce comparable shrimp microbiota (García-López et al., 2021). Thus, standard microbial community analyses are not overly sensitive to the particulars of binning approaches (Glassman and Martiny, 2018). In addition, we used a closed reference for taxonomical assignment, which has excluded spurious taxa. For these reasons, our findings are not biased by OTU clustering methods.

### Succession Pattern Between Bacterioplankton Community and Shrimp Gut Community

The diversity of shrimp gut microbiota was relatively stable, whereas the surrounding bacterioplankton diversity increased linearly during shrimp farming. Additionally, the diversity in gut microbiota was generally lower than that in corresponding bacterioplankton community (Supplementary Figure 1), in accordant with the notion that hosts select a subset of surrounding taxa that colonize into their gut (Zoqratt et al., 2018). In contrast, the linearly increased diversity of bacterioplankton could be attributed to the accumulation of nutritional sources along shrimp farming (Supplementary Figure 2), leading to the diversification of microbes. Both the shrimp gut microbiota and bacterioplankton community exhibited sequential changes over shrimp ontogeny (Figure 1). Consistently, there is ample evidence that the gut microbiota is primarily affected by host age in diverse aquatic animals (Burns et al., 2016; Yan et al., 2016). Given the sequential changes in both the gut microbiota and bacterioplankton communities (Figure 1), we compared temporal turnover rate of the two communities. Bacterioplankton community exhibited significantly steeper turnover (more rapid deviation from original to new state) than gut microbiota (Figure 2), indicating that host-associated communities are more temporally stable than free-living counterparts. This finding was further supported by the MRM model, revealing that the succession of gut microbiota (*R*^2^ = 0.21) were less explained by the shrimp age, compared with that of bacterioplankton community (*R*^2^ = 0.66) (Table 2). It seems that host gut offers a relatively stable microenvironment for commensals. Consistent with this assertion, it has been shown that fish gut is a more suitable environment than external skin mucus (Sylvain et al., 2020). In other words, gut microbiota experiences regular incremental shifts such as the maturity of physiology and immunity over shrimp ontogeny. Additionally, the rapid succession of bacterioplankton community could be attributed to temporally varied water geochemical variables during shrimp farming (Supplementary Figure 2), instead of direct role of shrimp age itself (see details in Figure 3), although we have tried to minimize colinearity among environmental factors. Indeed, the important role of shrimp age in governing both communities did not completely rule out the effects of other factors. We also detected that water temperature and pH strongly affected the temporal successions of total and abundant bacterial communities in both habitats but not the rare subcommunities (Table 2). Water temperature and pH have been extensively observed as key factors in shaping bacterioplankton community (Luo et al., 2019; Nyirabuhoro et al., 2019). Interestingly, shrimp are poikilotherms, while their gut microbiotas are sensitive to changes in rearing water temperature (23.8–30.7°C) (Supplementary Figure 2). Changes in the environmental temperature of aquaculture water could affect the metabolic and physiological functions of shrimp, thus indirectly alter the gut microbiotas. In accordance, it has been shown that water temperature affects feeding, growth, and survival of *Litopenaeus vannaei*. Rearing water temperature was positively associated with the abundances of anaerobes and the anaerobic Bifidobacterium (Li et al., 2018). Considering the functional importance of gut microbiota in host health, this pattern may partially explain why a sudden change in water temperature generally causes shrimp disease (Estrada-Perez et al., 2020). Furthermore, water temperature could directly alter the bacterioplankton community (Yang et al., 2018), which in turn affects shrimp gut microbiota. It is worthy to emphasize that lifestyle is also key factor in shaping the gut microbiota of shrimp (Cornejo-Granados et al., 2018). More specifically, there are distinct gut microbiotas between wild and aquacultured shrimp (Cornejo-Granados et al., 2017), low- and high-salinity–cultured shrimp (Hou et al., 2020), and freshwater and marine conditions (Cornejo-Granados et al., 2018). In this regard, follow-up investigations following a spatial sampling strategy is needed to test whether the pattern observed here is shared between culture ecosystems or ecosystem dependent.

### Interplay Among Geochemical Factor, Bacterioplankton Community, and Gut Microbiota Over Shrimp Ontogeny

SEM uncovered that rearing water salinity, pH, TP, and DO were the key determinants in driving the succession of bacterioplankton communities (Figure 3), corroborating recent studies obtained in shrimp, crab, and tilapia aquaculture conditions (Giatsis et al., 2015; Yang et al., 2018; Dai et al., 2020; Hou et al., 2020). Water DO, pH, and salinity directly affected the structures of bacterioplankton community (Figure 3), in accordance with the notion that bacterioplankton communities are extremely sensitive to subtle environmental changes (Or et al., 2012; Yang et al., 2018). In aquaculture ecosystem, the level of water phosphorus is usually low, thus bacterioplankton communities often experience P-unsaturation (Duhamel et al., 2021). Accordingly, the concentration of TP was significantly and positively correlated with the structures of bacterioplankton community (Liu et al., 2019). However, we found that a sharp increase in TP at the later farming stage exerted a negative effect on bacterioplankton communities (Figure 3 and Supplementary Figure 2). In contrast, there were negligible and insignificant effects of water salinity, pH, TP, and DO on the gut microbiota (Figure 3 and Supplementary Table 3). A possible explanation for this pattern is that host could buffer external environmental change. As a result, the gut microbiota is less affected by water geochemical factors. Bacterioplankton community only exerted a weak direct effect on the gut microbiota, compared with shrimp age (Figure 3). Consistently, ample evidence has shown that the gut microbiota in aquatic animals is distinct from surrounding environments (Zhang et al., 2018, 2021; Xiong et al., 2019b). It has been proposed that hosts selectively filter particular bacteria from the rearing environments, rather than randomly ingesting surrounding taxa (Stephens et al., 2016; Yan et al., 2016). In accordance, shrimp age (a proxy of gut maturity) is the main biological variable governing the succession of gut microbiota (Figures 2, 3 and Table 2). Together, changes in the geochemical variables strongly affect the structures of bacterioplankton community during shrimp farming, whereas the gut microbiota does not simply mirror the rearing bacterioplankton community.

### Shrimp Gut Commensals Sourced From Their Larvae

Although shrimp live in rearing water, relative abundances of the top 20 bacterial genera in shrimp gut were insignificantly associated (12 in 20 cases) with those in bacterioplankton community (Figure 4). For example, *Vibrio* genus was predominant in shrimp gut but was rare in rearing water. This is consistent with previous studies showing that the dominant genera are distinct between shrimp gut and rearing water and sediment (Zhou et al., 2021). *Vibrio* and *Photobacterium* members are long known to be opportunistic pathogens in shrimp aquaculture (Manilal et al., 2010). Nevertheless, the majority of vibrios are not pathogenic, many are commensal or even beneficial, including the carbon cycle and osmoregulation (Johnson, 2013). Indeed, *Vibrio* species have been frequently detected as a dominant population in shrimp gut (Chaiyapechara et al., 2012). In accordance, a few vibrio strains, e.g., *Vibrio alginolyticus* UTM 102, have been applied as probiotics in shrimp aquaculture (Balcázar et al., 2007). Similarly, *Photobacterium* strains are common in the intestinal contents of marine animals (Chaiyapechara et al., 2012). In addition, *Ruegeria*, *Marivita*, and *Flavobacterium* harbor the specific ability in degrading organic matter (Williams et al., 2012; Sun et al., 2019; Huang et al., 2020), which were enriched at the late stage of aquaculture water (Figure 4). The relative abundance of *Pseudoalteromonas* in shrimp gut was negatively associated with that in rearing water (Figure 4). Consistently, *Pseudoalteromonas* strains have been successfully used as probiotics in shrimp farming. In these regard, shrimp could select some beneficial commensals that improve their fitness.

Furthermore, we evaluated the contribution of internal sources (gut commensals of younger host) on the shrimp gut microbiota. Theoretically, aquatic animals are born without microorganisms, thus their gut commensals should source from the surrounding environments after birth (Yan et al., 2016). However, the majority of gut commensals of shrimp gut microbiota sourced from their younger host, rather than bacterioplankton communities (Figure 5), which reinforces the importance of the gut microbiome in younger host (Kerr et al., 2015). Similarly, it has been shown that shrimp acquires a small proportion of commensals from rearing water over development (Xiong et al., 2019b). The contribution of surrounding bacterioplankton communities on gut commensals markedly varied over shrimp ontogeny (Figure 5). We propose several explanations for this pattern. According to the co-evolution hypothesis (McFall-Ngai et al., 2013), it is mandatory for larva to recruit suitable taxa that expand the range of diet digestion due to incomplete digestive system. Thus, to improve hosts’ fitness, the colonization of gut commensals is filtered from rearing species pool as a result of deterministic processes. However, as host matures, the initial “winners” could be reassembled, thereby resulting in host stage-specific gut microbiota (Stephens et al., 2016; Yan et al., 2016). Additionally, temporal dynamics of environmental variables directly alter the bacterioplankton communities (Figure 4), leading to non-linear contribution of bacterioplankton communities to gut microbiota along shrimp farming. The skewed source pattern on day 56 could be induced by sudden increase in TP content and the low level of DO (Figure 2). In accordance, the SEM uncovered that water TP and DO exerted indirect effects on the gut microbiota (Supplementary Table 3). However, there were still a high proportion of “unknown” sources (Figure 5), which could be attributed to the uncollected species pools, e.g., diet, air and farmer. Together, gut commensals primarily source from adjacent younger shrimp. In this regard, we propose the isolation of probiotics from larval gut, which could be persist over shrimp ontogeny.

### Ecological Processes Governing the Assembly of Bacterial Community

Bacterioplankton communities are more closely phylogenetically clustered than the gut microbiotas, as supported by significantly lower mean value of ses.MNTD (Supplementary Figure 4), as observed in the present study and elsewhere (Xiong et al., 2019b). In addition, the βNTI values of gut microbiota and bacterioplankton community divergently changed during shrimp farming (Figures 6A,B), though both communities exhibited sequential shifts in the community structure (Figures 1, 2). The gut microbiota tended to be governed by variable selection (βNTI values > 2), while the bacterioplankton community was affected by homogeneous selection (βNTI values < –2) (Figures 6A,B). The logic behind this may be that shrimp has not reached full maturity, though we collected samples over an entire shrimp farming (Lucas et al., 2010). Consistent with this assertion, the gut microbiota significantly changed between every paired sampling (Supplementary Table 1). Similarly, it has been shown that the succession of shrimp gut microbiota is more driven by species replacement than bacterioplankton community (Xiong et al., 2019b). In contrast, geochemical variables of rearing water were relatively stable at the later farming days (Supplementary Figure 2), thus bacterioplankton communities were governed by homogeneous selection. Accordingly, the structures of bacterioplankton community were comparable between some adjacent pairs (Supplementary Table 2). Thus, host-associated and free-living bacterial communities are governed by different ecological processes. Considering the functional importance of gut microbiota in host health, additional works are required to explore the underlying ecological processes in governing the overlooked host-associated microbes. Notably, rare subcommunities in both the gut and rearing water were affected by homogeneous selection (Figures 6E,F), whereas their abundant counterparts were shaped by random processes (Figures 6C,D). Rare members serve as “seed bank” in a given community, which could switch to abundant taxa in response to changing environments (Magurran and Henderson, 2003). That is, rare taxa adapt to specific conditions that are strongly selected by external factors. In accordance, rare subcommunities were governed by deterministic processes (Figure 6). Corroborating recent works, rare subcommunity shown to be dominated by deterministic processes, while abundant subcommunity is influenced largely by stochastic processes in agricultural soils (Jiao and Lu, 2020) and freshwater ecosystems (Liu et al., 2015). The broad fitness of abundant taxa facilitates their successive establishment across a wide range of environmental conditions (Wan et al., 2021), e.g., variations in host maturity and geochemical factors here. By this logic, the abundant subcommunities are less affected by local variables, leading to the predominance of stochasticity (Figure 6). That is, no phylogenetic signs were detected for abundant communities. Consistently, there is ample evidence that rare taxa exhibit greater sensitivity to environmental factors than abundant species (Mo et al., 2018). Under these scenarios, it seems that the rare subcommunities are governed by deterministic processes, while the assembly of their abundant counterparts was stochastic across habitats, such as host gut and rearing water here.

## Conclusion

Host-associated bacterial community is more temporally stable than their free-living counterpart, as supported by the significant lower temporal turnover rate. In accordance, the gut microbiota is less affected by the rearing water geochemical variables, compared with bacterioplankton community. Intriguingly, the shrimp gut microbiota does not simply mirror the rearing bacterioplankton communities. Instead, gut commensals mainly inherit from their younger shrimp, rather than the rearing water. It seems that host-associated and free-living microbes are assembled by divergently ecological processes. That is, the gut microbiota is governed by variable selection over shrimp ontogeny, while the rearing bacterioplankton community is shaped by homogeneous selection. However, the determinism of rare and stochasticity of abundant subcommunities are consistent between shrimp gut and rearing water. These findings greatly broaden our understanding on the underlying ecological processes governing the temporal successions of host-associated and free-living microbial communities.

## Data Availability Statement

Raw sequence data are available in the BIG Data Center, Chinese Academy of Sciences, under code CRA004710 at http://bigd.big.ac.cn/gsa.

## Ethics Statement

The animal study was reviewed and approved by the Guide for the Care and Use of Laboratory Animals of Ningbo University.

## Author Contributions

JC and JX conceived and designed the research. WZ, ZZ, and QQ conducted the experiments. JX contributed analytical tools. JX and WZ analyzed the data. WZ wrote the manuscript with help from JX and ZZ. All authors read and approved the manuscript.

## Conflict of Interest

The authors declare that the research was conducted in the absence of any commercial or financial relationships that could be construed as a potential conflict of interest.

## Publisher’s Note

All claims expressed in this article are solely those of the authors and do not necessarily represent those of their affiliated organizations, or those of the publisher, the editors and the reviewers. Any product that may be evaluated in this article, or claim that may be made by its manufacturer, is not guaranteed or endorsed by the publisher.

## Footnotes

1
http://www.r-project.org
