# Supplementary material for: Quantifying the Importance of Abiotic and Biotic Factors Governing the Succession of Gut Microbiota Over Shrimp Ontogeny
Source: Front Microbiol. 2021 Oct 8;12:752750. doi: 10.3389/fmicb.2021.752750 (PMC8531273; doi:10.3389/fmicb.2021.752750)

**Frontier in Microbiology**

**Quantifying the Importance of Abiotic and Biotic Factors Governing the Succession of Gut Microbiota over Shrimp Ontogeny**

Wenqian Zhang ^1, 2^, Zidong Zhu ^3^, Jiong Chen ^1, 2^, Qiongfen Qiu ^2^, Jinbo Xiong ^1, 2, *^

^1^ State Key Laboratory for Managing Biotic and Chemical Threats to the Quality and Safety of Agro-products, Ningbo University, Ningbo 315211, China

^2^ School of Marine Sciences, Ningbo University, Ningbo 315211, China

^3^ School of Biochemical Engineering, Jingzhou Institute of Technology, Jingzhou 434020, China

***Corresponding author**

Jinbo Xiong, E-mail address: [xiongjinbo@nbu.edu.cn](mailto:xiongjinbo@nbu.edu.cn)

Tel.: 86-574-87608368; Fax: 86-574-87608347

**Table S1** Pairwise community dissimilarity test of the shrimp gut bacterial communities based on analysis of similarity (ANOSIM) using Bray-Curtis dissimilarity

|  | G7 | G14 | G21 | G28 | G35 | G43 | G49 | G56 | G70 | G77 | G87 | G93 |
| --- | --- | --- | --- | --- | --- | --- | --- | --- | --- | --- | --- | --- |
| G7 |  | 0.672 | 0.689 | 0.606 | 0.835 | 1.000 | 0.987 | 0.869 | 0.837 | 0.976 | 0.998 | 0.956 |
| G14 | **0.002** |  | 0.452 | 0.452 | 0.857 | 1.000 | 1.000 | 1.000 | 0.923 | 1.000 | 1.000 | 0.961 |
| G21 | **0.003** | **0.019** |  | 0.267 | 0.581 | 0.996 | 0.996 | 0.902 | 0.907 | 0.983 | 1.000 | 0.537 |
| G28 | **0.001** | **0.002** | **0.035** |  | 0.319 | 0.815 | 0.819 | 0.802 | 0.728 | 0.806 | 0.943 | 0.659 |
| G35 | **0.002** | **0.002** | **0.002** | **0.006** |  | 0.867 | 0.831 | 0.657 | 0.581 | 0.93 | 1.000 | 0.861 |
| G43 | **0.002** | **0.001** | **0.004** | **0.005** | **0.004** |  | 0.318 | 1.000 | 0.792 | 0.994 | 1.000 | 1.000 |
| G49 | **0.009** | **0.001** | **0.004** | **0.005** | **0.007** | **0.004** |  | 0.972 | 0.453 | 0.904 | 1.000 | 1.000 |
| G56 | **0.005** | **0.006** | **0.004** | **0.003** | **0.002** | **0.005** | **0.003** |  | 0.397 | 0.993 | 1.000 | 0.987 |
| G70 | **0.002** | **0.003** | **0.004** | **0.001** | **0.002** | **0.002** | **0.007** | **0.019** |  | 0.357 | 0.835 | 0.717 |
| G77 | **0.002** | **0.002** | **0.004** | **0.002** | **0.003** | **0.001** | **0.003** | **0.003** | **0.011** |  | 0.563 | 0.907 |
| G87 | **0.001** | **0.006** | **0.005** | **0.002** | **0.001** | **0.001** | **0.001** | **0.004** | **0.003** | **0.004** |  | 1.000 |
| G93 | **0.005** | **0.008** | **0.003** | **0.003** | **0.003** | **0.004** | **0.002** | **0.005** | **0.002** | **0.006** | **0.005** |  |

Top diagonal cells are r values, and lower diagonal cells are *P* values. Bold values represent significant differences (*P* < 0.05) between bacterial communities in pairs of sampling groups

**Table S2** Pairwise community dissimilarity test of the bacterioplankton communities based on analysis of similarity (ANOSIM) using Bray-Curtis dissimilarity

|  | W7 | W14 | W21 | W28 | W35 | W43 | W49 | W56 | W70 | W77 | W87 | W93 |
| --- | --- | --- | --- | --- | --- | --- | --- | --- | --- | --- | --- | --- |
| W7 |  | 0.331 | 0.981 | 0.969 | 1.000 | 0.998 | 1.000 | 1.000 | 1.000 | 0.946 | 1.000 | 1.000 |
| W14 | **0.014** |  | 0.522 | 0.624 | 0.874 | 0.893 | 1.000 | 0.989 | 1.000 | 0.939 | 1.000 | 1.000 |
| W21 | **0.003** | **0.009** |  | 0.166 | 0.706 | 0.987 | 1.000 | 1.000 | 1.000 | 0.969 | 1.000 | 1.000 |
| W28 | **0.002** | **0.003** | 0.072 |  | 0.415 | 0.933 | 0.989 | 0.969 | 0.998 | 0.874 | 1.000 | 0.989 |
| W35 | **0.003** | **0.003** | **0.003** | **0.002** |  | 0.852 | 0.963 | 0.822 | 0.985 | 0.783 | 0.998 | 0.906 |
| W43 | **0.003** | **0.003** | **0.005** | **0.001** | **0.004** |  | 0.364 | 0.659 | 0.9 | 0.737 | 0.987 | 0.922 |
| W49 | **0.004** | **0.005** | **0.002** | **0.003** | **0.002** | **0.003** |  | 0.11 | 0.744 | 0.631 | 0.893 | 0.759 |
| W56 | **0.002** | **0.001** | **0.005** | **0.004** | **0.002** | **0.001** | 0.183 |  | 0.468 | 0.341 | 0.669 | 0.641 |
| W70 | **0.004** | **0.002** | **0.005** | **0.003** | **0.002** | **0.003** | **0.005** | **0.003** |  | 0.103 | 0.485 | 0.439 |
| W77 | **0.003** | **0.004** | **0.003** | **0.003** | **0.003** | **0.004** | **0.002** | **0.018** | 0.152 |  | 0.292 | 0.294 |
| W87 | **0.003** | **0.001** | **0.002** | **0.004** | **0.004** | **0.002** | **0.003** | **0.004** | **0.006** | **0.015** |  | 0.091 |
| W93 | **0.002** | **0.003** | **0.001** | **0.001** | **0.003** | **0.004** | **0.003** | **0.003** | **0.005** | **0.005** | 0.123 |  |

Top diagonal cells are r values, and lower diagonal cells are *P* values. Bold values represent significant differences (*P* < 0.05) between bacterial communities in pairs of sampling groups.

**Table S3** Direct and indirect relationships between variables. The path coefficients are calculated by SEM after 999 bootstraps.

|  | Estimate | Std. Error | | t value | Pr(>\|t\|) |
| --- | --- | --- | --- | --- | --- |
| Salinity → BC | 0.28 | 0.10 | 2.69 | | 0.007 |
| pH → BC | 0.23 | 0.08 | 3.06 | | 0.002 |
| DO → BC | 0.14 | 0.07 | 1.98 | | 0.048 |
| TP → BC | -0.34 | 0.07 | -4.69 | | < 0.001 |
| Age → Salinity | 0.80 | 0.07 | 10.98 | | < 0.001 |
| Age → pH | 0.55 | 0.10 | 5.55 | | < 0.001 |
| Age → DO | 0.41 | 0.11 | 4.81 | | < 0.001 |
| Age →TP | 0.50 | 0.10 | 4.81 | | < 0.001 |
| Age → BC | 0.50 | 0.12 | 4.09 | | < 0.001 |
| Age → GM | 0.34 | 0.16 | 2.12 | | 0.034 |
| GM→ AGM | 0.91 | 0.05 | 17.84 | | < 0.001 |
| GM → RGM | 0.39 | 0.11 | 3.50 | | < 0.001 |
| BC → ABC | 0.89 | 0.05 | 16.54 | | < 0.001 |
| BC → RBC | 0.46 | 0.11 | 4.30 | | < 0.001 |
| BC → GM | 0.13 | 0.16 | 0.79 | | 0.43 |
| Total effects |  |  |  | |  |
| Relationships | Direct | Indirect | Total | |  |
| Salinity → BC | 0.28 | 0 | 0.28 | |  |
| pH → BC | 0.23 | 0 | 0.23 | |  |
| DO → BC | 0.14 | 0 | 0.14 | |  |
| TP → BC | -0.34 | 0 | -0.34 | |  |
| Age → Salinity | 0.80 | 0 | 0.80 | |  |
| Age → pH | 0.55 | 0 | 0.55 | |  |
| Age → DO | 0.41 | 0 | 0.41 | |  |
| Age →TP | 0.50 | 0 | 0.50 | |  |
| Age → BC | 0.50 | 0.24 | 0.74 | |  |
| Age → GM | 0.34 | 0.09 | 0.43 | |  |
| GM → AGM | 0.91 | 0 | 0.91 | |  |
| GM → RGM | 0.39 | 0 | 0.39 | |  |
| BC → ABC | 0.89 | 0 | 0.89 | |  |
| BC → RBC | 0.46 | 0 | 0.46 | |  |
| BC → GM | 0.13 | 0 | 0.13 | |  |
| DO → GM | 0 | 0.02 | 0.02 | |  |
| pH → GM | 0 | 0.03 | 0.03 | |  |
| Salinity → GM | 0 | 0.04 | 0.04 | |  |
| TP → GM | 0 | -0.01 | -0.04 | |  |

BC: Bacterioplankton community; GM: Gut microbiota; AGM: Abundant gut microbiota; RGM: Rare gut microbiota; ABC: Abundant bacterioplankton community; RBC: Rare bacterioplankton community; WT: water temperature; DO: dissolved oxygen; TP: total phosphorus.

**Fig. S1** Comparisons of bacterial α-diversity between shrimp gut and the corresponding rearing water at each life stage and at the two locations using paired t test. Cycles and triangles represent rearing water and shrimp gut samples, respectively. NS: not significant; *: P < 0.05; **: P < 0.01.


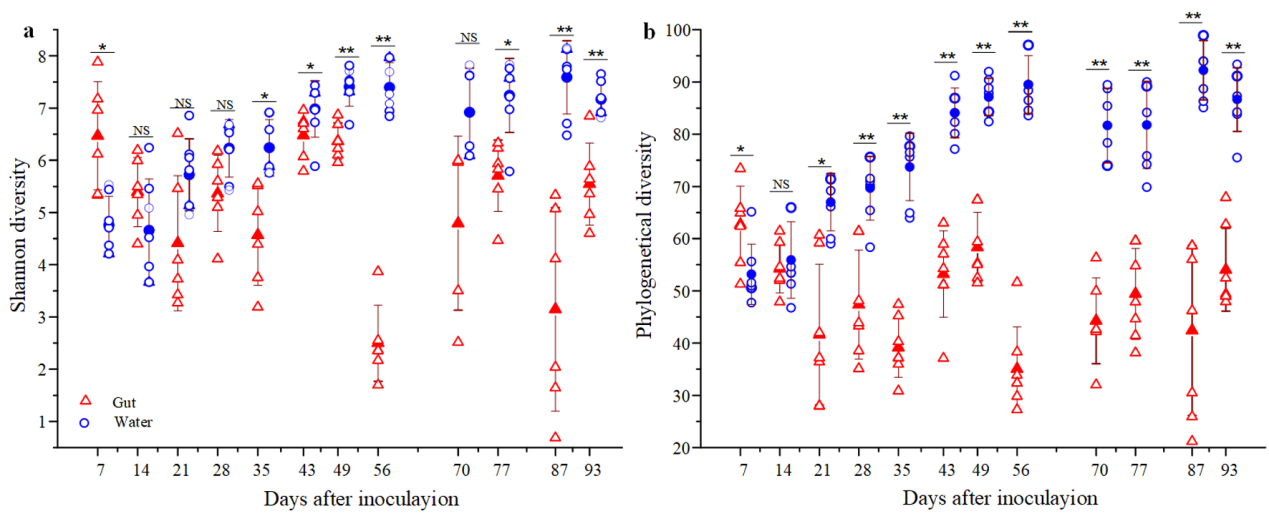


**Fig. S2** Changes in water environmental factors over time.


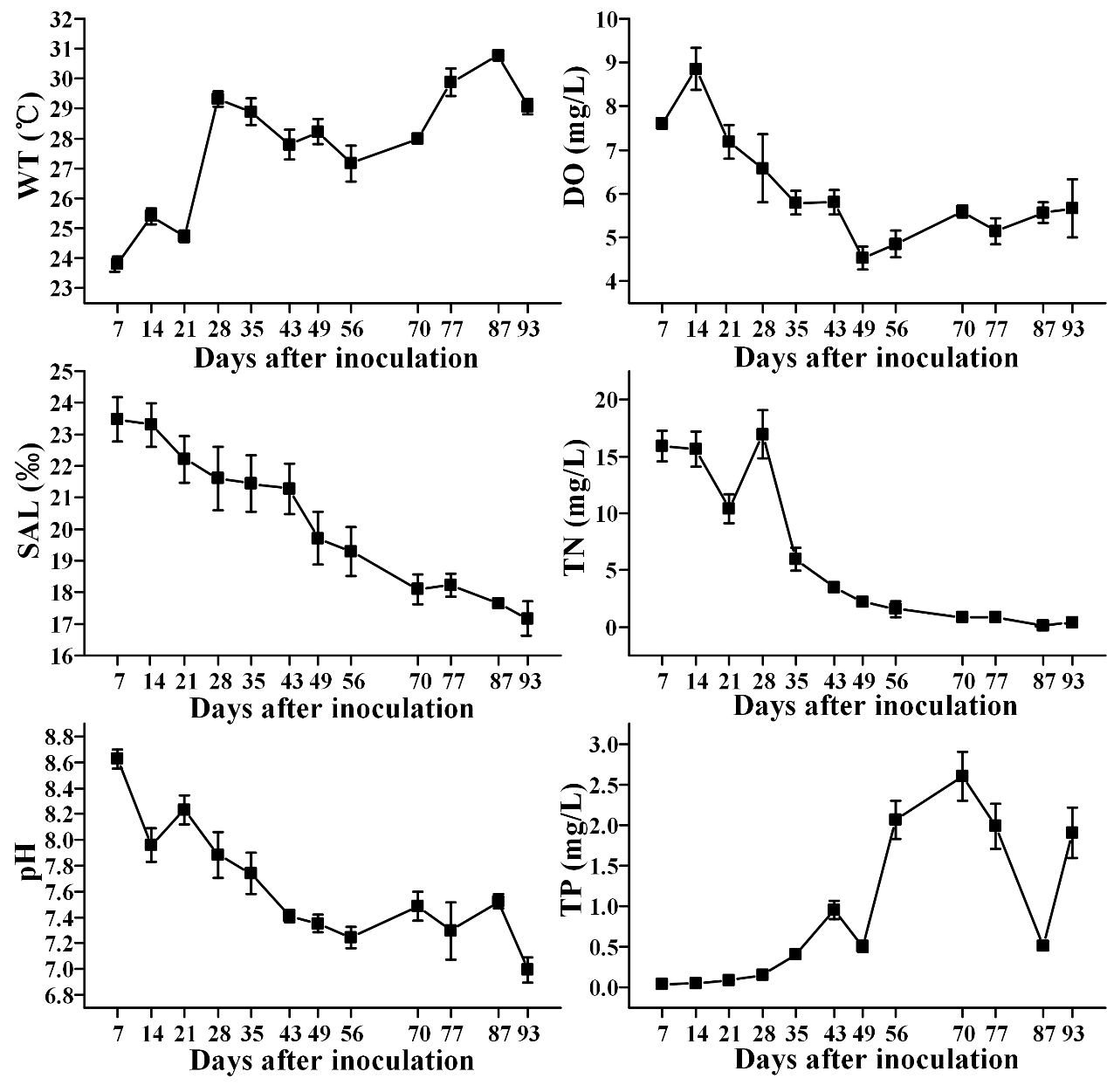


WT: water temperature; DO: dissolved oxygen; SAL: salinity; TN: total nitrogen; TP: total phosphorus **Fig. S3** Venn diagram shows the unique and shared OTUs between shrimp gut and rearing water on each sampling day.


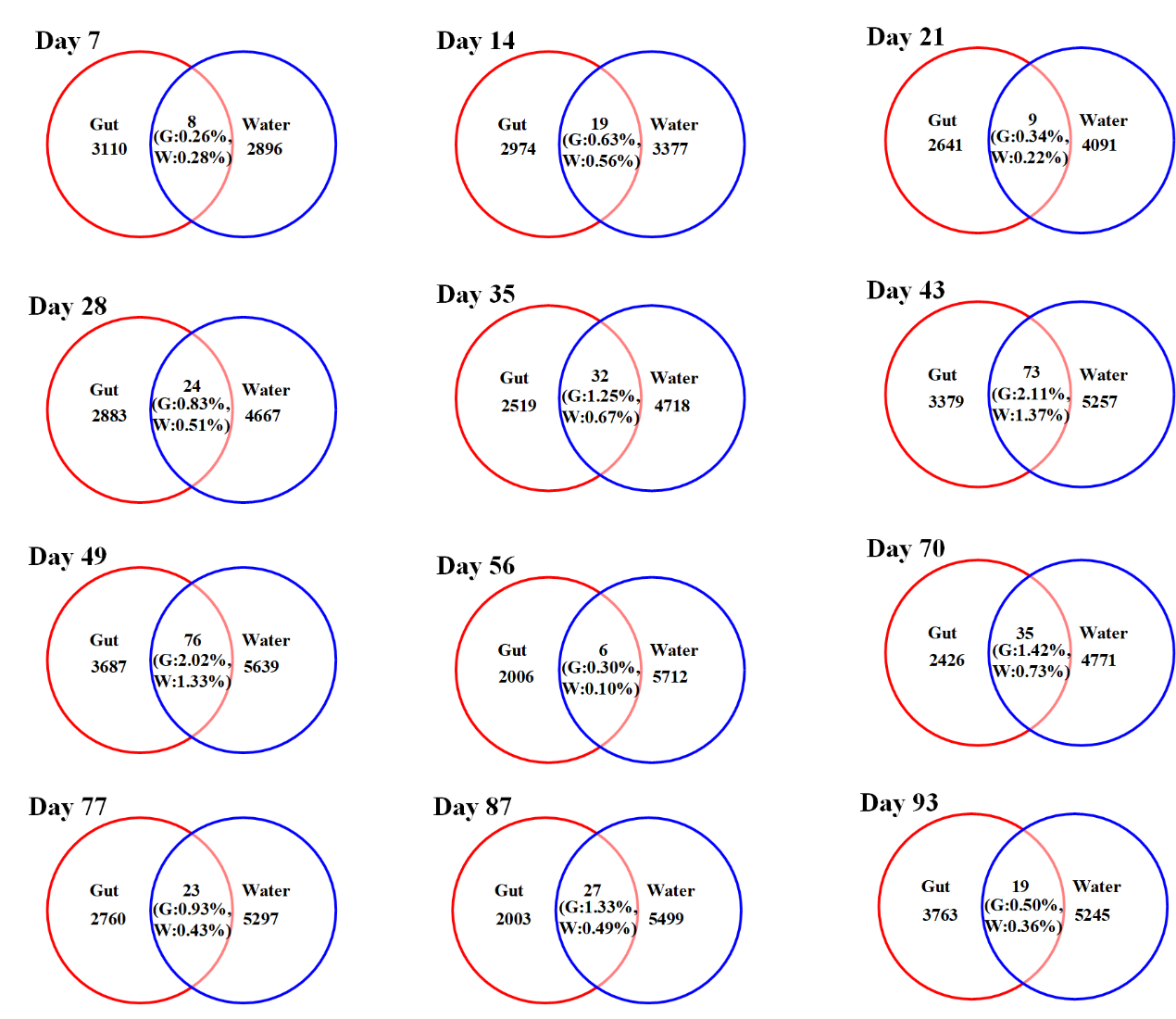
**Fig. S4** The boxplot of community ecological processes of communities in shrimp gut and rearing water. The dashed line denotes equal roles for both. An individual community below the line indicates that determinism dominantly governs the community assembly, while a community above the line indicates that stochasticity is dominant. The different lowercase letters denote significant differences among the different community based on a one-way ANOVA. Patterns of ses.MNTD across days for gut microbiota (a-c) and bacterioplankton community (d-f). Different letters indicate statistically significant at *P* < 0.05.


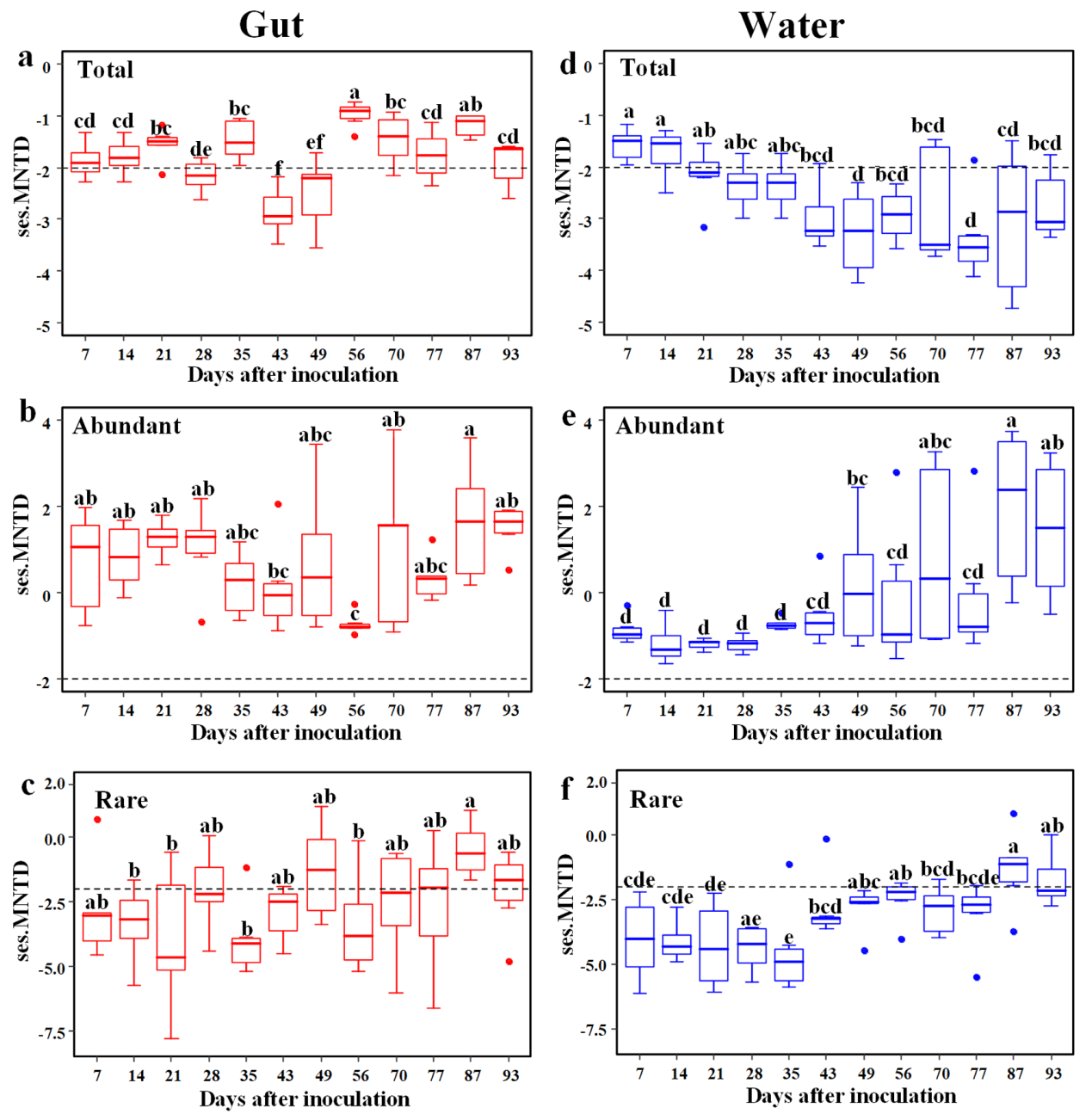


**Fig. S5** Beta nearest taxon index (βNTI) value under different communities. The horizontal dashed line indicate the βNTI values of ﹣2 and +2.The different lowercase letters denote significant differences among the different community based on a one-way ANOVA. Patterns of βNTI across days for gut microbiota (a-c) and bacterioplankton community (d-f). Different latters indicate statistically significant at *P* < 0.05.


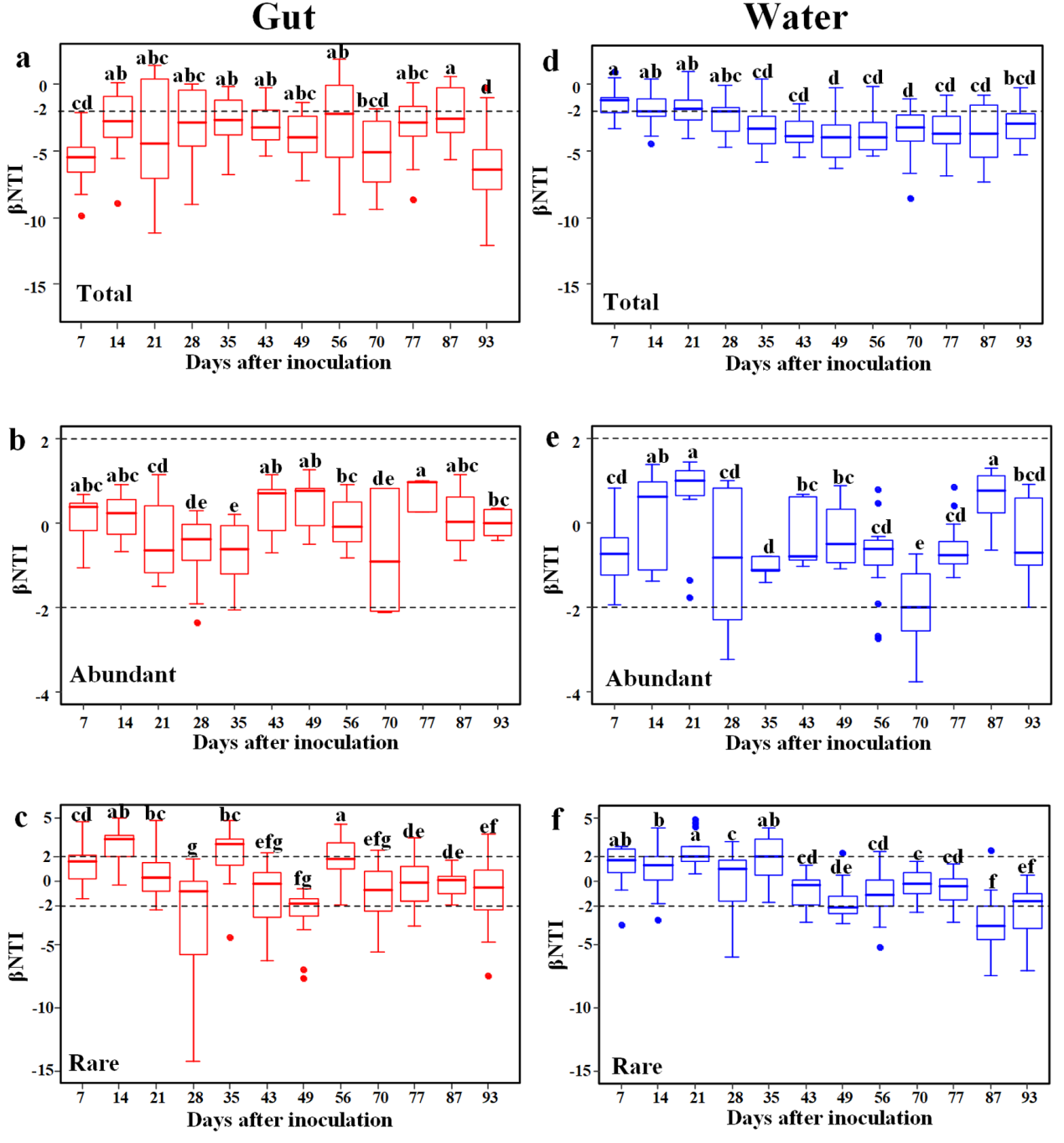

Supplement: Supplementary file 1 [file Data_Sheet_1.docx]
